# Can physical exercise alleviate social anxiety in junior high school students by improving body image?

**DOI:** 10.3389/fpsyg.2026.1780759

**Published:** 2026-03-11

**Authors:** Suxuan Xing, Yang Yang, Jingtao Wu, Yanhong Shao, Wanli Zang

**Affiliations:** 1 School of Sports Training, Chengdu Sport University, Chengdu, China; 2 School of Physical Education, Leshan Normal University, Leshan, China; 3 Xiangshui Teacher Development Center, Yancheng, China; 4 Postgraduate School, Harbin Sport University, Harbin, China

**Keywords:** body image, cross-lagged model, longitudinal study, mental health, physical exercise, social anxiety

## Abstract

**Objective:**

This study aims to investigate the dynamic relationships between physical exercise (PE), body image (BI), and social anxiety (SA) among Chinese junior high school students, as well as to analyze the mechanism through which PE may alleviate SA by improving BI.

**Methods:**

A total of 851 students (652 males, 391 females) from grades 7 and 8 across five secondary schools in Sichuan, Jiangsu, and Guangdong provinces were selected for this 12-month longitudinal study. Four waves of data were collected using validated questionnaires measuring PE, BI, and SA. The data were analyzed using a random intercept cross-lagged panel model (RI-CLPM).

**Results:**

The study revealed the following findings: (1) PE showed significant positive correlations with BI at all four time points (*r* = 0.308 to 0.463, *p* < 0.001) and significant negative correlations with SA (*r* = −0.142 to −0.301, *p* < 0.001); (2) BI was also significantly negatively correlated with SA (*r* = −0.138 to −0.313, *p* < 0.001); (3) PE significantly reduced subsequent SA levels by improving BI. The total effect of the pathway PE(T1) → BI(T2) → SA(T3) was −0.301, with a mediation effect of −0.125 (accounting for 41.53% of the total effect).

**Conclusion:**

This study elucidates the mediating mechanism through which PE alleviates SA by enhancing BI, providing scientific evidence for adolescent mental health interventions. Schools are advised to incorporate strategies in physical education curricula and activities that foster positive BI and social adaptation skills to promote psychological well-being.

## 1 Introduction

Adolescence is a critical period for psychological development, during which social anxiety (SA) emerges as a prevalent mental health concern. With the development of internet technology, SA among adolescents has garnered increasing scholarly attention in recent years (Beesdo et al., 2009; Xian et al., 2024). Scholars define SA as an individual’s experience of tension, unease, and avoidance behaviors in social situations due to fear of negative evaluation (Stein and Stein, 2008; Avramchuk, 2018). In adolescents, SA not only adversely affects academic performance and social interaction skills but also increases susceptibility to depression and feelings of loneliness (Hamed and Abbas, 2019; Spence and Rapee, 2016). During junior high school, adolescents face multifaceted challenges, including heightened academic pressure, restructuring of social relationships, and rapid physical development, all of which contribute to a marked increase in the prevalence of SA (Maes et al., 2019; Cannon et al., 2020). Consequently, identifying influencing factors and elucidating underlying mechanisms of SA have become pivotal topics in contemporary psychological and educational research.

Body image (BI), defined as an individual’s subjective evaluation and perception of their physical appearance, body shape, and physical competence, constitutes a significant factor influencing adolescent SA (Gan and Jiang, 2024; Marengo et al., 2018). A negative BI predisposes adolescents to heightened sensitivity toward others’ evaluations, thereby exacerbating SA (Moafi et al., 2019; Lin et al., 2024). Adolescence represents a critical period for BI concerns, as rapid physical development, heightened appearance-related preoccupation, and increased social comparison tendencies contribute to more frequent negative BI experiences (Dorčić et al., 2023; Littleton, 2008). Within Chinese cultural contexts, deeply ingrained societal ideals favoring slender body types and an emphasis on academic achievement may further reinforce adolescents’ negative self-perceptions and body-related anxieties (Stojcic et al., 2020; Ren et al., 2018). Conversely, a positive BI enhances self-confidence, reduces excessive preoccupation with others’ judgments, and significantly mitigates SA (Yixin et al., 2024; Jia, 2025). To date, however, the precise mechanisms through which BI influences SA remain insufficiently understood, warranting further empirical investigation.

Physical exercise (PE) is widely recognized as a common intervention for student mental health, with scholars generally agreeing that it serves as an important means of improving BI and alleviating SA (Jia, 2025; Warburton and Bredin, 2017). Regular physical activity enhances body composition, boosts physical fitness, and promotes physiological mechanisms such as endorphin release, all of which contribute to improved psychological states and significant reductions in SA (Rodriguez-Ayllon et al., 2019; Stonerock et al., 2015). In group-based sports activities, increased social interaction enhances adolescents’ social adaptability, indirectly strengthening self-efficacy and progressively mitigating SA (Teixeira et al., 2024; Wu et al., 2025). However, adolescents with high SA often exhibit heightened concerns about BI, leading to avoidance of PE and trapping them in a vicious cycle of “low exercise–high anxiety” (Chen et al., 2024; Walder et al., 2025). While PE can improve BI and reduce SA, it may also be subject to reverse influences, necessitating further clarification of this bidirectional relationship.

Despite existing research elucidating the associations among PE, BI, and SA, several gaps remain in understanding their underlying mechanisms. First, most studies have been limited to cross-sectional designs, lacking longitudinal investigations into the dynamic interactions among these variables across multiple time points (Baribeau et al., 2022; Zika and Becker, 2021). Given that adolescent psychological development is characterized by distinct phases and is highly susceptible to sociocultural influences, cross-sectional analyses alone are insufficient for interpreting the internal relationships among these factors (Moafi et al., 2019). Second, the majority of research has been conducted in Western cultural contexts, with relatively few studies examining these relationships within China’s unique sociocultural and educational environment. In China, societal emphasis on thinness and academic achievement may reduce adolescents’ opportunities, time, and motivation for PE, exacerbating negative BI perceptions (Stojcic et al., 2020; Liu et al., 2025). Furthermore, the mediating role of BI and the potential suppressive effects of SA remain underexplored, warranting further investigation (Neill et al., 2020).

To address these gaps, this study employs a longitudinal design to systematically examine the dynamic relationships among PE, BI, and SA. Using a random intercept cross-lagged panel model (RI-CLPM), we analyze causal pathways and bidirectional mechanisms across four waves of data collected over 12 months from junior high school students. By incorporating demographic variables (e.g., gender, age, grade, and regional background), we assess the predictive effects of PE on BI and SA, as well as the mediating role of BI. This study aims to enrich theoretical frameworks in adolescent mental health research and provide evidence-based insights for educational interventions, psychological support, and physical activity programs, ultimately helping adolescents mitigate SA and enhance psychological well-being and social adaptability.

### 1.1 Relationship between PE and SA

The positive effects of PE in alleviating anxiety symptoms among adolescents have been well-documented in numerous studies, establishing it as a significant mental health intervention strategy (Ewuzie et al., 2024; Hoare et al., 2016). Physical activity directly mitigates anxiety by reducing physiological arousal levels, promoting endorphin secretion, and enhancing stress regulation capabilities (Warburton and Bredin, 2017; Herring et al., 2010). Regular aerobic exercise has been consistently associated with marked reductions in anxiety levels among adolescents (Szuhany et al., 2025). In SA contexts, PE further improves emotional states by enhancing physical health and athletic performance (Zhang et al., 2024). Notably, exercise participation significantly boosts adolescents’ self-confidence in their abilities while decreasing sensitivity to negative evaluations (Eime et al., 2013). Adolescents engaged in physical activities demonstrate greater social initiative and lower avoidance behaviors in interpersonal interactions (Zhao et al., 2025), suggesting that exercise may improve social functioning through increased self-confidence and reduced anticipatory anxiety. However, current research has predominantly focused on generalized anxiety reduction, leaving the psychological mechanisms underlying exercise effects in specific SA contexts insufficiently explored, particularly across diverse cultural backgrounds (Uchino et al., 2018).

Team-based sports hold particular significance for developing adolescents’ social competencies and appear especially effective in mitigating SA (Neill et al., 2020). These activities not only provide enhanced opportunities for social interaction but also foster a sense of belonging and achievement through collective cooperation and goal attainment (Uchino et al., 2018; Jiang et al., 2025). Adolescents participating in team sports like basketball and football exhibit superior social adaptation skills and lower SA levels (Zika and Becker, 2021; Eime et al., 2013). This phenomenon aligns with social support theory, which posits that team activities reduce interpersonal stress by facilitating positive peer interactions and support networks (Cohen, 2014). Furthermore, team sports enhance satisfaction with peer relationships and social belonging—key factors in SA reduction (Sabiston et al., 2019). However, individual experiences in team settings may vary considerably depending on personality traits, participation frequency, and team dynamics (Pilkionienė et al., 2021). Introverted adolescents may experience heightened psychological pressure in team environments, potentially attenuating the anxiety-reducing benefits of exercise (Wang et al., 2023). Future investigations should examine these moderating factors more thoroughly and develop tailored intervention strategies for different personality types.

SA itself may create a self-perpetuating cycle by limiting exercise participation, resulting in a “high anxiety-low exercise” feedback loop (Wu et al., 2025). Adolescents with elevated SA tend to avoid situations involving potential evaluation, including physical activities and team sports (Zuckerman et al., 2021). This avoidance behavior deprives individuals of opportunities for mental health improvement through exercise, consequently exacerbating social apprehension and discomfort (Wang and Liu, 2022). Some highly anxious individuals may specifically avoid athletic settings due to performance-related concerns (Jiang et al., 2025). This bidirectional relationship suggests that exercise and SA interact dynamically, influenced by both psychological characteristics and social contexts (Buchan et al., 2021). Current research recommends designing more inclusive and supportive exercise environments for socially anxious individuals, such as small-group activities or non-competitive formats (Wang et al., 2023). Longitudinal study designs could further elucidate temporal effects and causal pathways between exercise and SA, informing evidence-based psychological intervention strategies (Miers et al., 2020).

### 1.2 Relationship between BI and SA

BI refers to an individual’s subjective assessment and perceptual experience of their own physical appearance and bodily functions. During adolescence, BI cognition exerts a significant impact on psychological states and emotional stability (Revranche et al., 2022). Existing studies have consistently confirmed a significant positive correlation between negative BI and SA (Moafi et al., 2019). Dissatisfaction with one’s appearance exacerbates concerns about others’ negative evaluations, thereby increasing avoidance behaviors and elevating anxiety levels (Zhao et al., 2024). Research indicates that adolescents overly focused on weight issues or self-perceived appearance flaws tend to face more social difficulties and exhibit avoidance behaviors (Xiang et al., 2024). This internalized sense of insecurity distorts the processing of social information, strengthens persistent SA, and forms a vicious cycle (King et al., 2020). It is thus evident that BI plays a crucial role in adolescent SA and merits in-depth exploration.

Within the Chinese cultural context, the association between BI and SA may present more complex patterns. Unlike Western aesthetic standards, contemporary Chinese society advocates for a slim physique and academic achievement, which exacerbates adolescents’ negative self-evaluation of their appearance (Ren et al., 2018). Compared with Western adolescents, Chinese adolescents show greater concern about their appearance, especially during pubertal development, which significantly hinders normal social interactions (Wang et al., 2024). Those with higher appearance anxiety display more cautious and timid behaviors in public settings and group activities (Liu et al., 2025). Furthermore, society’s emphasis on academic performance reduces adolescents’ opportunities to participate in physical activities, which may further aggravate SA (Abdoli et al., 2024). Although these cultural factors clearly influence the formation of SA, their underlying mechanisms remain to be explored in depth.

Notably, there may be a bidirectional relationship between BI and SA. Individuals with higher levels of SA tend to reduce social interactions due to excessive fear of negative evaluations, thereby accumulating more negative perceptions of BI (Gunnell et al., 2016). Adolescents with significant SA exhibit more severe social avoidance behaviors (Feng et al., 2024), accompanied by stronger appearance-related rumination, which further reduces the positivity of BI (Wang et al., 2024). As scholars have pointed out, negative BI may exacerbate SA by increasing sensitivity to others’ evaluations and fear of social failure (Kajastus et al., 2024). Improving adolescents’ BI may alleviate their subsequent levels of SA, and vice versa (Niven et al., 2009). Therefore, future research should adopt longitudinal designs and dynamic analysis methods to systematically examine this dialectical relationship, providing a scientific basis for psychological interventions and policy-making targeting adolescent populations.

### 1.3 The impact of PE on BI

PE, defined as regular engagement in sporting activities, is widely acknowledged by scholars as an effective approach to enhancing body morphology and a primary intervention for adolescent mental health (Kostanski et al., 2004). Through systematic physical activity, exercise refines body shape, boosts physical fitness, and reinforces functional body awareness, thereby substantially elevating satisfaction with one’s physical appearance (Hale et al., 2021). Research has indicated that consistent physical activity can effectively alleviate adolescents’ negative emotions related to body management while enhancing self-confidence (Choukas-Bradley et al., 2022). Rooted in self-identity theory, PE fosters positive assessments of bodily capacities and enhances overall appearance perception (Revranche et al., 2022). Exercise offers adolescents a constructive setting that restores functional body cognition and alleviates appearance-related anxieties (Liu et al., 2025).

In team sports activities, adolescents gain not only a sense of achievement through peer interaction and goal accomplishment but also strengthen positive BI perceptions via affirming social feedback (Jazaieri et al., 2012). Individuals participating in team sports (e.g., football or basketball) are more prone to experiencing team support and recognition, where such positive social interactions markedly enhance appearance satisfaction (Abdoli et al., 2024). The supportive social milieu in team activities cultivates a sense of belonging, enhances BI, and diminishes appearance-related anxiety (Ren et al., 2018). Notably, this effect is particularly pronounced among female adolescents, given their heightened sensitivity to appearance evaluations, rendering them more likely to develop body confidence through exercise (Plotnikoff et al., 2013). However, this benefit may be moderated by individual personality traits, exercise frequency, and team dynamics, as introverted adolescents may derive less positive feedback from team interactions (Chiu et al., 2021).

Although PE demonstrates significant benefits for BI improvement, this process may exhibit bidirectionality influenced by initial BI status (Straatmann et al., 2019). Adolescents with poor BI may avoid sports participation due to fear of judgment, consequently diminishing exercise’s potential benefits (Doré et al., 2016). Those with high appearance anxiety tend to shun body-revealing activities (e.g., swimming or running) (Chen et al., 2024), with such avoidance behaviors further undermining exercise motivation. A dynamic interaction exists between BI improvement and PE: positive exercise experiences enhance BI, while improved BI subsequently increases exercise participation (O'gorman et al., 2020).

### 1.4 Interactive relationships among PE, BI, and SA

Intricate connections and mechanisms link PE, BI, and SA. Exercise might indirectly ease SA by enhancing body morphology (Deng and Wang, 2024). Consistent exercise involvement positively affects satisfaction with body shape and functional awareness, notably boosting social confidence while lowering sensitivity to negative judgments (Choukas-Bradley et al., 2022). Aerobic workouts improve appearance perception, reduce social panic and timidity, and alleviate SA (Chen et al., 2024), implying BI’s potential mediating role in the exercise-SA link.

Conversely, SA may hinder exercise participation, diminishing exercise’s positive impacts on BI (Maes et al., 2019). Adolescents with high SA often feel inferior and avoid social interactions, especially in team activities where excessive fear of mockery triggers negative appraisals and avoidance behaviors (Kostanski et al., 2004). Such avoidance further lessens motivation and chances to engage in group sports, worsening SA. These individuals face a “high anxiety-low exercise” cycle that impairs emotional stability, exacerbates negative BI, and blocks anxiety relief (Sabiston et al., 2019). Future interventions should thus include non-competitive, low-exposure activity plans with virtual or small-group exercises to reduce participation avoidance (Chen et al., 2024).

The tripartite relationship among PE, BI, and SA likely involves bidirectional and dynamic interactions requiring longitudinal investigation (Zika and Becker, 2021). Positive BI may alleviate SA through confidence enhancement, encouraging active sports participation that further reduces anxiety (Jia, 2025). Additionally, cultural context may significantly moderate these interactions. In Chinese culture that idealizes slimness, appearance anxiety may particularly influence exercise’s effect on SA (Xiang et al., 2024). Future research should adopt multicultural longitudinal designs with random intercept cross-lagged modeling to systematically examine dynamic trajectories and causal pathways, providing scientific basis for intervention strategies.

### 1.5 Research gaps and study rationale

While existing studies have examined the relationships between PE, BI, and SA, several critical limitations remain: First, the predominant use of cross-sectional designs in current research precludes causal inferences and obscures the dynamic interplay among these variables. Second, the specific mechanisms through which PE may alleviate SA via BI improvement remain insufficiently validated, particularly among adolescent populations in the Chinese cultural context. Furthermore, the potential bidirectional relationship whereby SA may conversely inhibit exercise participation has received inadequate empirical attention.

**Figure 1:**
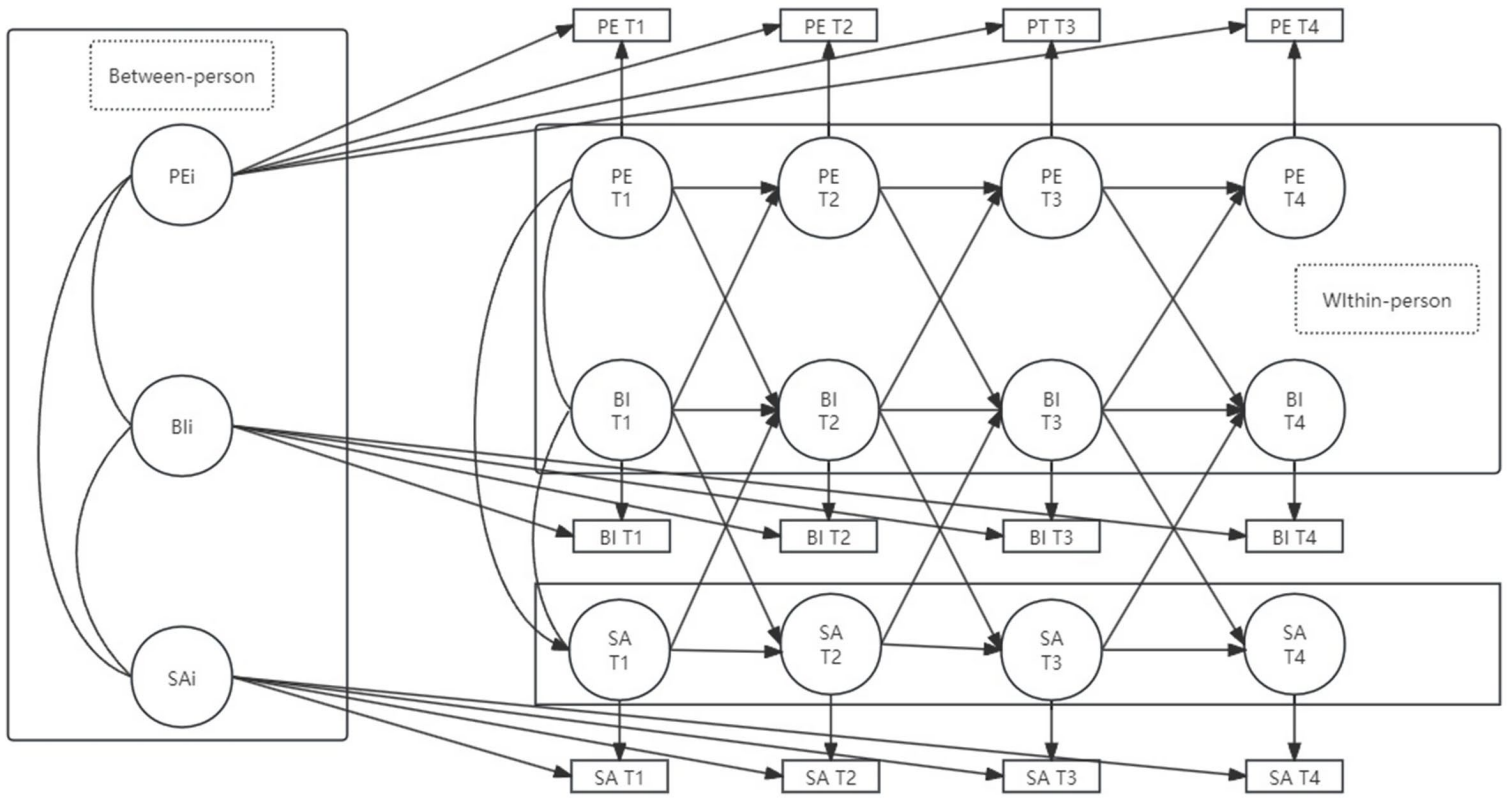
Research hypothesis.

To address these gaps, the present study employs a rigorous longitudinal design featuring a random intercept cross-lagged panel model (RI-CLPM) to systematically investigate the dynamic interactions among PE, BI, and SA in Chinese junior high school students, while controlling for key demographic variables. Three primary hypotheses guide this investigation: (H1) PE can significantly negatively predict SA in the next period; (H2) BI will mediate the relationship between PE and SA; (H3) SA will reciprocally inhibit exercise participation behaviors, demonstrating a bidirectional dynamic relationship. Figure 1 presents the conceptual model visually depicting these hypothesized relationships.

## 2 Methods

### 2.1 Participants

This study employed a cluster sampling method to recruit seventh, eighth, and ninth-grade students from five secondary schools in Leshan City (Sichuan Province), Yancheng City (Jiangsu Province), and Guangzhou City (Guangdong Province) for a four-wave longitudinal survey. The baseline data collection commenced in September 2023, with three subsequent waves conducted at approximately 3-month intervals over a 12-month period. The study protocol was approved by the Institutional Review Board (Approval No.: LSNU:1034–24-12RO), with written informed consent obtained from participating schools and parents. Prior to each survey, participants were briefed on research objectives and confidentiality protocols, with voluntary participation and anonymous responses ensured.

The initial wave distributed 1,426 questionnaires, yielding 1,043 valid responses (response rate: 73.2%), including 652 males (62.51%) and 391 females (37.49%). Inclusion criteria required participants to: (1) be currently enrolled in junior high school; (2) possess independent questionnaire-completion ability; and (3) exhibit no significant physical or mental health impairments. Valid response rates for subsequent waves were 967 (retention rate: 92.71%), 896 (92.66%), and 851 (91.95%), respectively. The final sample (N = 851) had a mean age of 14.62 years (SD = 0.48), with males comprising 63.57% and females 36.43%. Age distribution showed 89.19% of participants aged 12–14 years.

The student sample was predominantly composed of eighth graders (86.37%), with smaller proportions from the seventh (4.2%) and ninth (8.81%) grades. This uneven distribution suggests that the findings are most applicable to early-to-middle adolescents. Notably, higher attrition rates were observed in the seventh and ninth grades, likely because seventh graders are adjusting to a new school environment while ninth graders are preoccupied with graduation examinations. Rural students slightly outnumbered urban peers (52.06% vs. 47.94%). Participating schools represented mid-to-upper socioeconomic status (SES), with 58% having at least one parent with tertiary education (slightly below national average). Rural household registration prevalence (19%) exceeded urban national averages. No significant differences emerged across waves in gender ratios, key variables, or scale scores (*p* > 0.05). Attrition analysis revealed no significant differences between completers and dropouts on primary measures (*p* > 0.05).

### 2.2 Procedure

The data collection for this study spanned 1 year and was conducted in four phases at approximately three-month intervals, with the initial phase commencing in September 2023. All data were collected in a classroom setting during students’ concentrated learning sessions, with the research team providing on-site guidance and technical support. To minimize measurement bias, questionnaires were administered in randomized order, and data collection followed a standardized protocol (Waaktaar et al., 2025).

The measurement tools included the International Physical Activity Questionnaire for Adolescents (IPAQ-A), the BI Scale for Adolescents (BISA), and the SA Scale for Adolescents (SAS-A). These instruments demonstrated good reliability and validity, effectively assessing adolescents’ physical activity levels, BI, and SA. The questionnaire completion time was controlled within 20–30 min, and all data were collected anonymously. To enhance participants’ focus, detailed explanations of the research purpose, instructions, and confidentiality assurances were provided before each data collection session. Upon completion, the research team conducted double-checking and cleaning of the questionnaires, excluding invalid responses and coding valid data for analysis (Sabiston et al., 2019).

SPSS 26.0 and Mplus 8.0 were employed for statistical modeling and path analysis. Specifically, a Random Intercept Cross-Lagged Panel Model (RI-CLPM) was used to explore the dynamic relationships between PE, BI, and SA, examining temporal effects and causal pathways (Kajastus et al., 2024). The analysis controlled for potential confounding variables such as gender, age, and geographical origin. Retention rate analysis and attrition tests were performed across multiple time points, confirming that sample attrition did not significantly affect the study’s conclusions (Wang et al., 2023). After the study, the team provided feedback to schools and participants in the form of reports and mental health education manuals, along with evidence-based recommendations for mental health improvement.

### 2.3 Measurement instruments

#### 2.3.1 Physical activity scale

The International Physical Activity Questionnaire for Adolescents (IPAQ-A) was used to assess participants’ physical activity levels. Developed by an international collaborative research team, the IPAQ-A demonstrates strong reliability and validity (Hagströmer et al., 2006). The adolescent version consists of seven items evaluating the frequency and duration of moderate-intensity, vigorous-intensity physical activity, and walking. Responses were scored on a 7-point scale, with total scores ranging from 0 to 28; higher scores indicate greater physical activity levels. The model exhibited good fit indices (*χ*^2^/df = 3.78, CFI = 0.91, GFI = 0.91, RMSEA = 0.058, SRMR = 0.05). Internal consistency reliability (Cronbach’s *α*) across the four time points was 0.88, 0.86, 0.84, and 0.82, indicating high consistency and longitudinal stability. Maximum likelihood estimation was applied, with Satorra-Bentler correction (S-B *χ*^2^) to address potential non-normality (Satorra and Bentler, 2001).

#### 2.3.2 BI scale

The BI Scale for Adolescents (BISA) was employed to evaluate participants’ BI perceptions. The BISA has demonstrated strong psychometric properties and cross-cultural applicability (O’Dea, 2004). It comprises 15 items across two dimensions: Body Appearance Satisfaction (BAS) and Body Function Perception (BFP), assessing cognitive and affective attitudes toward physical form and functionality. Items were rated on a 5-point Likert scale (1 = *strongly disagree* to 5 = *strongly agree*), with total scores ranging from 15 to 75; higher scores reflect more positive BI. The bifactor model showed excellent fit (*χ*^2^/df = 3.45, CFI = 0.94, GFI = 0.93, RMSEA = 0.055, SRMR = 0.045). Cronbach’s *α* coefficients across the four waves were 0.87, 0.89, 0.88, and 0.85, confirming high reliability and longitudinal stability. Maximum likelihood estimation with Satorra-Bentler correction was applied to address non-normality (Satorra and Bentler, 2001).

#### 2.3.3 SA scale

The SA Scale for Adolescents (SAS-A), developed by Finnish scholars, was used to assess SA levels. The SAS-A exhibits robust validity, reliability, and cross-cultural suitability (La Greca and Lopez, 1998). It includes 18 items across three subscales: Fear of Negative Evaluation (FNE), Social Avoidance and Distress (SAD), and Anxiety in New Social Situations (ANS). Responses were recorded on a 5-point Likert scale (1 = *not at all* to 5 = *extremely*), with total scores ranging from 18 to 90; higher scores indicate greater SA. The three-factor model demonstrated good fit (*χ*^2^/df = 3.65, CFI = 0.93, GFI = 0.92, RMSEA = 0.057, SRMR = 0.05). Internal consistency (Cronbach’s *α*) across the four time points was 0.89, 0.91, 0.88, and 0.86, indicating strong longitudinal stability. Maximum likelihood estimation with Satorra-Bentler correction was used to address non-normality (Satorra and Bentler, 2001).

### 2.4 Statistical analysis

Data analysis was performed using SPSS 27.0 and Mplus 8.3. A Random Intercept Cross-Lagged Panel Model (RI-CLPM) was employed to examine the dynamic relationships between PA, BI, and SA. This approach disentangles time-invariant components (random intercepts) from time-varying components (cross-lagged effects), controlling for stable between-individual differences while focusing on within-person dynamics. Unlike the traditional cross-lagged model, RI-CLPM captures time-varying trends in both between-person and within-person differences, thereby offering a more complete representation of longitudinal dynamics (Hamaker et al., 2015). Data from all four measurement waves were included, with gender incorporated as a covariate to assess autoregressive effects (stability over time) and cross-lagged effects (predictive relationships between variables).

$Y_{t}+1_{\mathrm{ij}}=\alpha_{j}+\beta_{1}Y_{\mathrm{tij}}+\beta_{2}X_{\mathrm{tij}}+\beta_{3}Z_{\mathrm{tij}}+\beta_{4}{\text{GENDER}}_{\mathrm{ij}}+\varepsilon_{\mathrm{ij}}$

Where *Yt* + 1, *ijYt*+1, *ij* represents the dependent variable (e.g., SA) at time *t* + 1 *t* + 1 for individual i*i* in class *jj*, *αjαj* denotes the random intercept for class *jj* to account for between-class variability, *β*1 captures the autoregressive effect (stability of the dependent variable over time), *β*2 and *β*3 represent cross-lagged effects (predictive relationships between PA, BI, and SA), *β*4 controls for the effect of gender, and *εijεij* is the residual error term. Model fit was assessed using multiple indices, including chi-square (*χ*^2^), comparative fit index (CFI > 0.90), Tucker-Lewis index (TLI > 0.90), root mean square error of approximation (RMSEA < 0.08), and standardized root mean square residual (SRMR < 0.08) (Kline, 2023). Results are presented as unstandardized coefficients to ensure interpretability of effect sizes.

## 3 Results

### 3.1 Correlation matrix

As presented in Table 1, all variables demonstrated significant autocorrelations across time points, indicating substantial temporal stability. PE showed autocorrelations ranging from 0.318 to 0.463 (*p* < 0.001), BI ranged between 0.334 and 0.457 (*p* < 0.001), and SA exhibited autocorrelations from 0.294 to 0.332 (*p* < 0.001). The cross-variable correlation analysis revealed three significant and theoretically meaningful patterns: (1) PE showed a robust positive correlation with BI (*r* = 0.308–0.463, *p* < 0.001); (2) PE was negatively correlated with SA (*r* = −0.142 to −0.301, *p* < 0.001); and (3) BI and SA exhibited a consistent negative relationship (*r* = −0.138 to −0.313, *p* < 0.001). The study confirmed the establishment of the H1 and H2 research hypotheses, that is, there is a significant positive correlation between PE and BI, and BI is negatively correlated with SA.

**Table 1:** Correlation matrix of observed variables across periods.

	1	2	3	4	5	6	7	8	9	10	11	12
PE(T1)	1											
PE(T2)	0.396^**^	1										
PE(T3)	0.390^**^	0.411^**^	1									
PE(T4)	0.318^**^	0.422^**^	0.463^**^	1								
BI(T1)	0.449^**^	0.401^**^	0.393^**^	0.317^**^	1							
BI(T2)	0.379^**^	0.455^**^	0.389^**^	0.403^**^	0.421^**^	1						
BI(T3)	0.373^**^	0.416^**^	0.460^**^	0.447^**^	0.405^**^	0.426^**^	1					
BI(T4)	0.308^**^	0.421^**^	0.441^**^	0.453^**^	0.334^**^	0.431^**^	0.457^**^	1				
SA(T1)	−0.301^**^	−0.142^**^	−0.189^**^	−0.178^**^	−0.299^**^	−0.138^**^	−0.177^**^	−0.156^**^	1			
SA(T2)	−0.212^**^	−0.289^**^	−0.167^**^	−0.162^**^	−0.204^**^	−0.286^**^	−0.156^**^	−0.168^**^	0.294^**^	1		
SA(T3)	−0.251^**^	−0.265^**^	−0.306^**^	−0.165^**^	−0.245^**^	−0.250^**^	−0.291^**^	−0.154^**^	0.235^**^	0.320^**^	1	
SA(T4)	−0.198^**^	−0.232^**^	−0.187^**^	−0.297^**^	−0.204^**^	−0.235^**^	−0.192^**^	−0.313^**^	0.332^**^	0.322^**^	0.321^**^	1

Physical exercise, PE; body image, BI; *p < 0.05. social anxiety, SA; **p < 0.001. T1, Time 1; T2, Time 2; T3, Time 3; T4, Time 4.

### 3.2 Longitudinal measurement invariance testing

As shown in Table 2, the longitudinal measurement invariance tests confirmed that all three constructs—PE, BI, and SA—demonstrated satisfactory measurement invariance across time points. For PE, all three levels of invariance (configural, metric, and scalar) showed excellent model fit (*χ*^2^/df = 2.35–2.65, RMSEA ≤ 0.02, CFI ≥ 0.94) with acceptable differences between nested models (ΔRMSEA ≤ 0.001, ΔCFI ≤ 0.002).

**Table 2:** Longitudinal measurement invariance tests.

Variable	Type	*x*^2^/df	RMSEA	CFI	ΔRMSEA	ΔCFI
PE	Configural	2.35	0.01	0.94	–	–
	Metric	2.25	0.02	0.95	0.001	0.001
	Scalar	2.65	0.01	0.94	0.001	0.002
BI	Configural	1.86	0.01	0.96	–	–
	Metric	1.88	0.02	0.95	0.002	0.001
	Scalar	1.96	0.03	0.95	0.001	0.004
SA	Configural	4.32	0.02	0.96	–	–
	Metric	4.42	0.04	0.95	0.001	0.003
	Scalar	4.51	0.02	0.95	0.002	0.005

ΔRMSEA indicates the change in Root Mean Square Error of Approximation following model adjustment, while ΔCFI represents the change in Comparative Fit Index after model modification.

Similarly, BI exhibited strong measurement invariance with good model fit across all levels (*χ*^2^/df = 1.86–1.96, RMSEA≤0.03, CFI ≥ 0.95) and minimal differences between models (ΔRMSEA ≤ 0.002, ΔCFI ≤ 0.004). SA also achieved full measurement invariance, demonstrating acceptable fit indices (*χ*^2^/df = 4.32–4.51, RMSEA ≤ 0.04, CFI ≥ 0.95) and meeting all criteria for model comparisons (ΔRMSEA ≤ 0.002, ΔCFI ≤ 0.005).

### 3.3 Cross-lagged effects of PE, BI, and SA

The cross-lagged model demonstrated acceptable fit indices (*χ*^2^/df = 8.54–38.53, CFI = 0.85–0.87, RMSEA = 0.02–0.04), supporting the overall model structure. Model comparisons revealed no significant difference between the constrained model (M2, with equal cross-lagged paths) and the freely estimated model (M1) (Δ*χ*^2^/df = 1.60, *p* = 0.261; ΔCFI = −0.01; ΔRMSEA = 0.02), indicating temporal consistency in cross-lagged effects. However, subsequent constraints on autoregressive paths (M3), correlated paths (M4), and all paths (M5) resulted in significant model deterioration (M3-M2: Δ*χ*^2^/df = 4.38, *p* < 0.001; M4-M3: Δ*χ*^2^/df = 8.12, *p* < 0.001; M5-M4: Δ*χ*^2^/df = 15.89, *p* < 0.001), suggesting time-specific variations in certain paths while maintaining overall model adequacy (see Table 3).

**Table 3:** Model fit and comparison for cross-lagged analysis of PE and SA.

Model	Model fit			Model comparison				
	*x*^2^/df	CFI	RMSEA [90%CI]		Δ*x*^2^/df	*P*	ΔCFI	ΔRMSEA
M1	8.54	0.87	0.02 [0.01, 0.05]		–	–	–	–
M2	10.14	0.86	0.04 [0.02, 0.05]	M2-M1	1.60	0.261	−0.01	0.02
M3	14.52	0.85	0.04 [0.02, 0.05]	M3-M2	4.38	<0.001	−0.01	0.00
M4	22.64	0.86	0.04 [0.02, 0.05]	M4-M3	8.12	<0.001	0.01	0.00
M5	38.53	0.87	0.04 [0.02, 0.05]	M5-M4	15.89	<0.001	0.01	0.00

M1 is the freely estimated model; M2 is the model with equality constraints on cross-lagged path coefficients across time; M3 is the model with equality constraints on autoregressive path coefficients across time; M4 is the model with equality constraints on correlation path coefficients across time; M5 is the model with equality constraints on all path coefficients across time. ΔRMSEA represents the change in the adjusted root mean square error of approximation (RMSEA), and ΔCFI represents the change in the adjusted comparative fit index (CFI).

**Figure 2:**
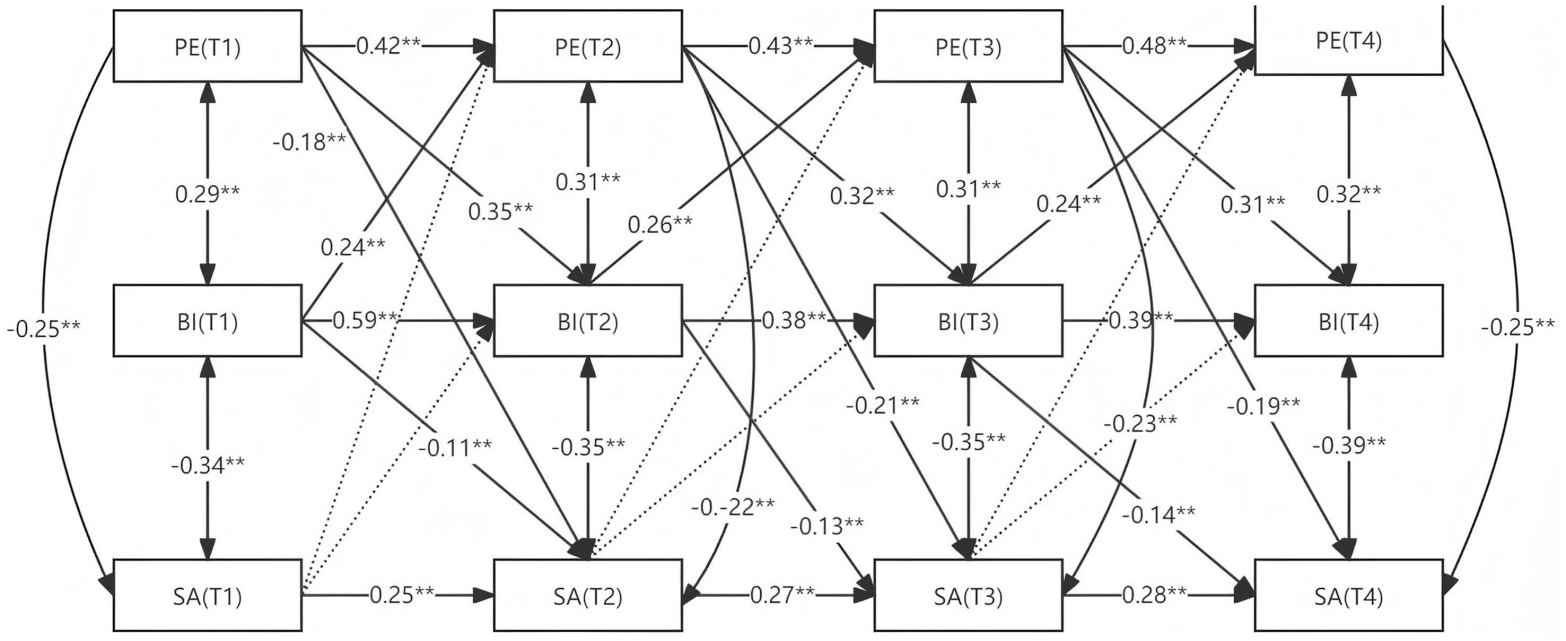
Cross-lagged model of PE, BI, and SA.

As detailed in Figure 2 and Table 4, PE exerted significant indirect effects on SA through BI. The total effect of PE(T1) → BI(T2) → SA(T3) was −0.301 (*p* < 0.001), with a mediation effect of −0.135 accounting for 44.94% of the total effect. Similarly, the PE (T2) → BI (T3) → SA (T4) pathway showed a mediation effect of −0.060 (*p* < 0.001, 25.99% of total effect), while the PE (T1) → BI (T3) → SA (T4) pathway demonstrated a mediation effect of −0.057 (*p* < 0.001, 25.98% of total effect). These findings consistently indicate that PE reduces subsequent SA through improvements in BI, with BI serving as a robust mediator in this longitudinal relationship.

**Table 4:** Test of the cross-lagged mediating effects.

Mediation pathway	Effect value				95% confidence interval		Proportion of effect
	Total effect	Indirect effect	Direct effect	*SE*	Lower limit	Upper limit	
PE(T1) → BI(T2) → SA(T3)	−0.301^**^	−0.135^**^	−0.166^**^	0.020	−0.341	−0.261	44.94%
PE(T2) → BI(T3) → SA(T4)	−0.232^**^	−0.060^**^	−0.172^**^	0.015	−0.262	−0.202	25.99%
PE(T1) → BI(T3) → SA(T4)	−0.228^*^	−0.057^**^	−0.171^**^	0.014	−0.255	−0.201	25.98%

Physical exercise, PE; body image, BI, *p < 0.05. social anxiety, SA; **p < 0.001. T1, Time 1; T2, Time 2; T3, Time 3; T4, Time 4.

### 3.4 Testing for mediation effects in random-intercept cross-lagged models

Table 5 indicates that the random-intercept cross-lagged model exhibits good fit (M1: *χ*^2^/df = 7.23, CFI = 0.91, RMSEA = 0.02). The model with constrained equal cross-lagged path coefficients (M2) shows no significant difference in fit compared to the freely estimated model (M1) (Δ*χ*^2^/df = 1.44, *p* = 0.154), supporting the consistency of cross-lagged paths across different time periods. However, when further constraining autoregressive path coefficients (M3), correlation path coefficients (M4), and all path coefficients (M5), the differences in model fit become progressively significant (M3 vs. M2: Δ*χ*^2^/df = 3.78, *p* < 0.001; M4 vs. M3: Δ*χ*^2^/df = 5.87, *p* < 0.001; M5 vs. M4: Δ*χ*^2^/df = 10.33, *p* < 0.001). This suggests that some path coefficients vary over time, though the overall fit indices (CFI ≥ 0.83, RMSEA ≤ 0.05) remain within acceptable ranges.

**Table 5:** Fit and comparison of random-intercept cross-lagged models for PE and SA.

Model	model fit			Model comparison				
	*x*^2^/df	CFI	RMSEA [90%CI]		Δ*x*^2^/df	*P*	ΔCFI	ΔRMSEA
M1	7.23	0.91	0.02 [0.01, 0.04]		–	–	–	–
M2	8.67	0.89	0.03 [0.02, 0.07]	M2-M1	1.44	0.154	−0.02	−0.001
M3	12.45	0.88	0.04 [0.02, 0.08]	M3-M2	3.78	<0.001	−0.01	−0.001
M4	18.32	0.86	0.04 [0.02, 0.08]	M4-M3	5.87	<0.001	−0.02	0.001
M5	28.65	0.83	0.05 [0.03, 0.08]	M5-M4	10.33	<0.001	−0.03	0.001

M1 is the freely estimated model; M2 is the model with equality constraints on cross-lagged path coefficients across time; M3 is the model with equality constraints on autoregressive path coefficients across time; M4 is the model with equality constraints on correlation path coefficients across time; M5 is the model with equality constraints on all path coefficients across time. ΔRMSEA represents the change in the adjusted root mean square error of approximation (RMSEA), and ΔCFI represents the change in the adjusted comparative fit index (CFI).

**Figure 3:**
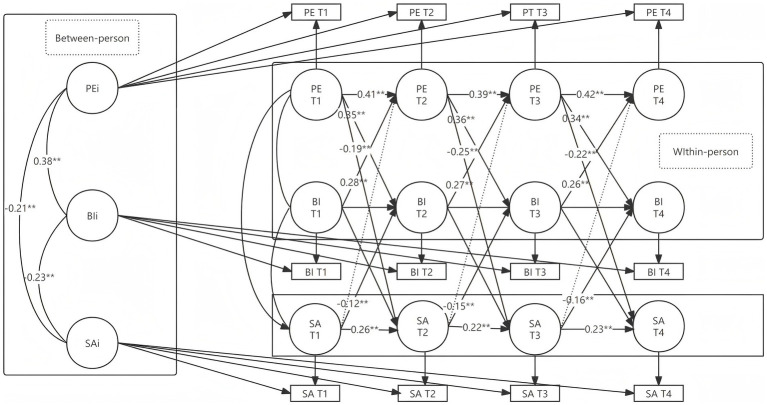
Random-intercept cross-lagged model of PE, BI, and SA.

Further analyses in Figure 3 and Table 6 reveal that PE exerts a significant indirect effect on SA through improving BI. For the pathway PE (T1) → BI (T2) → SA (T3), the total effect is −0.301 (*p* < 0.001), with a mediation effect of −0.125, accounting for 41.53% of the total effect. For PE (T2) → BI (T3) → SA (T4), the mediation effect is −0.055 (*p* < 0.001), contributing 23.71% to the total effect. For PE (T1) → BI (T3) → SA (T4), the mediation effect is −0.052 (*p* < 0.001), representing 22.81% of the total effect. These findings demonstrate that PE can indirectly reduce SA by enhancing BI, with BI playing a crucial mediating role. The study proves the establishment of H1, H2 and H3, that is, the RI-CLPM model is established under the individual differences, and PE is affected by BI to establish the SA effect, and SA inhibits sports participation inversely, showing a two-way dynamic interaction.

**Table 6:** Testing for cross-lagged mediation effects.

Mediation Pathway	Effect value				95% confidence interval		Proportion of effect
	Total effect	Indirect effect	Direct effect	*SE*	Lower limit	Upper limit	
PE(T1) → BI(T2) → SA(T3)	−0.301^**^	−0.125^**^	−0.176^**^	0.022	−0.344	−0.258	41.53%
PE(T2) → BI(T3) → SA(T4)	−0.232^**^	−0.055^**^	−0.177^**^	0.017	−0.265	−0.199	23.71%
PE(T1) → BI(T3) → SA(T4)	−0.228^*^	−0.052^**^	−0.176^**^	0.016	−0.260	−0.196	22.81%

Physical exercise, PE; body image, BI, *p < 0.05; social anxiety, SA; **p < 0.001; T1, Time 1; T2, Time 2; T3, Time 3; T4, Time 4.

## 4 Discussion

This study examined the complex longitudinal relationships among PE, BI, and SA (SA), emphasizing the critical role of BI as a mediating variable. PE was significantly positively correlated with BI at all time points and significantly negatively correlated with SA, while all three variables exhibited high temporal stability. Both the traditional cross-lagged model and the random-intercept cross-lagged model supported the key mediating role of BI in the process by which PE influences SA. The mediating effect of BI accounted for 41.53 to 44.94% of the total effect and remained significant across different time periods. Results of the longitudinal measurement invariance test indicated consistency in the cross-temporal measurement of each variable, enhancing the reliability of the study conclusions. This research provides further empirical support for the psychological benefits of PE and offers a new pathway for intervening in SA.

### 4.1 The mediating role of BI in the influence of PE on SA

Findings from the correlation coefficient matrix reveal that, across all time points, PE and BI maintain a significant positive correlation. In contrast, both PE and SA, as well as BI and SA, exhibit significant negative correlations—consistent with prior research (Lubans et al., 2016). Existing studies have shown that physical activity can effectively ease anxiety by enhancing BI, boosting individuals’ body satisfaction, and fostering self-efficacy. Notably, the consistent, stable positive association between PE and BI suggests that PE may serve as a key driver of improved BI, with such enhancements potentially playing an indirect role in reducing SA. Furthermore, results from the longitudinal invariance test (see Table 2) confirm the measurement consistency of PE, BI, and SA across temporal dimensions, laying a solid theoretical foundation for exploring dynamic inter-variable relationships.

The cross-lagged effect model was employed to further clarify the internal mechanisms linking PE, BI, and SA. A significant mediation effect was observed for the path PE (T1) → BI (T2) → SA (T3), as detailed in Table 2. This indicates that PE not only directly reduces anxiety but also indirectly alleviates SA by enhancing BI. This aligns with Bandura’s self-efficacy theory (1977, 1986, 1997) (Locke, 1997), which posits that individuals can strengthen confidence in their abilities through positive behavioral experiences (e.g., PE), thereby lowering anxiety levels. The study highlights that improving BI yields important psychological benefits for adolescents and young adults, echoing existing theoretical frameworks.

The mediating role of body image (BI) varies across developmental stages. This shift may reflect differences in individuals’ psychological needs across developmental stages. Early adolescents typically focus more on appearance and BI (Estrada-Tenorio et al., 2020), making BI improvements particularly critical for alleviating SA during this period. As individuals mature, other social factors (e.g., interpersonal relationships, academic pressure) may gradually become primary influencers of anxiety. Additionally, social support and cultural context may play important roles in regulating the relationship between PE and mental health (Babic et al., 2014), warranting further exploration in future studies. Overall, using longitudinal data and cross-lagged models, this study validates the hypothesis that PE reduces SA by enhancing BI, offering new perspectives and evidence for research in this domain (Cash and Fleming, 2002).

### 4.2 The stability of PE, BI, and SA

Longitudinal invariance test results (see Table 2) indicate that PE, BI, and SA exhibit favorable fit indices across configural, metric, and scalar invariance models (*χ*^2^/df = 1.86 to 4.51, RMSEA ≤ 0.04, CFI ≥ 0.94). Meanwhile, discrepancies between distinct models (ΔRMSEA ≤ 0.002, ΔCFI ≤ 0.005) fall within acceptable bounds, signifying strong measurement consistency among the three variables. Aligned with prior research outcomes (Lubans et al., 2016), the association between PE and BI persists, and both BI and individual anxiety levels demonstrate stability across varying time points.

Despite the high overall stability, analyses from the cross-lagged models (Tables 4, 5) reveal dynamic changes in the relationships among the three variables over time. For instance, although the positive impact of PE on BI and the negative impact on SA persist across time, the proportion of their mediating effects is not entirely consistent. The mediating effect of BI was significant in different periods, but it showed a decreasing trend with time. Such fluctuations may reflect shifts in individuals’ psychosocial needs at different developmental stages (Estrada-Tenorio et al., 2020). For example, in early adolescence, individuals are more sensitive to BI, making the positive effect of PE on BI more pronounced. As adolescence progresses into its middle and late stages or early adulthood, individuals’ focus may gradually shift to other social needs (such as academic pressure or interpersonal relationships), thereby weakening this mediating effect.

In addition, changes in SA may be closely related to an individual’s social environment and experiences. For example, accumulated social feedback or negative social situations (such as rejected or failed intimate interactions) can significantly influence anxious emotions (Locke, 1997). Although PE, BI, and SA exhibit high overall stability, their dynamic relationships may be affected by an individual’s developmental stage and social context. These potential factors require further exploration in future studies to more comprehensively unravel the complex interaction mechanisms among the three variables.

### 4.3 The mediating role of BI in the relationship between PE and SA and its cross-temporal consistency

The correlation coefficient matrix (Table 1) reveals a significant positive association between PE and BI, alongside a significant negative correlation between PE and SA. Moreover, BI and SA exhibit a significant negative linkage. These outcomes suggest that PE not only directly lessens SA but may also indirectly ease anxious feelings by enhancing BI. This aligns with prior research (Lubans et al., 2016), which states that physical activity effectively reduces BI-related anxiety by boosting individuals’ physical self-assurance. Consistent with Bandura’s (1997) self-efficacy theory (Locke, 1997), when people improve their BI through PE, they also strengthen their sense of competence and confidence, thereby further mitigating SA.

Table 4 further confirms the mediating role of BI in the relationship between PE and SA. The indirect pathway through which PE impacts SA via BI is significant across multiple time points. Overall, the mediation effect remained stable over the longitudinal period, though its magnitude fluctuated without a consistent directional trend. This variation indicates that the indirect influence of PE on SA fluctuates across different time points. Such fluctuations may relate to shifts in individuals’ psychological needs during various developmental stages. Early adolescents tend to focus more on appearance and BI (Field et al., 2001); thus, enhancing BI through PE has a more notable effect during this period, thereby alleviating anxiety more effectively. However, as individuals grow older, they may encounter mounting academic pressure, career demands, and interpersonal challenges. These social factors may gradually weaken the indirect effect of PE on reducing anxiety through BI.

Results from the random-intercept cross-lagged model (Table 5) further support the cross-temporal consistency among PE, BI, and SA. Fit tests indicate that the difference in fit between the model with constrained equal cross-lagged path coefficients (M2) and the freely estimated model (M1) is non-significant, suggesting high stability of the cross-lagged paths among the three variables across the time dimension. Meanwhile, the positive effect of PE on BI and the negative effect of BI on SA remain significant over time. For instance, the path coefficients of PE(T1) → BI(T2) and BI(T2) → SA(T3) show minimal variation. This aligns with the understanding that the positive impacts of PE on BI and mental health are long-term and stable (Littleton, 2008). Nevertheless, despite the overall consistency of the paths, the fluctuations in effect intensity over time warrant further investigation.

Furthermore, individuals’ social experiences and environmental factors may exert important influences on the dynamic changes in the relationships among the three variables. For example, negative social feedback or setbacks in intimate relationships may directly exacerbate anxious emotions, thereby weakening the indirect effect of PE on alleviating SA through improved BI. Changes in SA may also be affected by individuals’ subjective evaluation of whether their BI conforms to social standards (Alcaraz-Ibáñez et al., 2023). Therefore, although the mechanisms among PE, BI, and SA exhibit a certain degree of cross-temporal consistency, their dynamic changes may be jointly regulated by factors such as individuals’ developmental stages, social feedback, and cultural backgrounds. Future research could further explore the specific roles of these moderating variables.

### 4.4 Limitations

Despite revealing the dynamic relationships among PE, BI, and SA through cross-lagged models and a longitudinal design, this study has several limitations. First, the sample is primarily drawn from adolescents in specific regions, and cultural backgrounds and social support may limit the generalizability of the results (Estrada-Tenorio et al., 2020). The uneven sampling across grades suggests that the conclusion may be more applicable to early to middle adolescence. Second, although multi-time-point measurements enhance the stability of the study, the time span is relatively short, failing to capture longer-term trends. Additionally, as an observational study, causal inferences must be made cautiously; potential confounding variables such as social support, academic pressure, or family environment may impact the results (Hollon and Sexton, 2012). Regarding measurement tools, the assessment of BI focuses mainly on appearance satisfaction, failing to comprehensively cover functional dimensions (Frederick and Reynolds, 2022). Future research could incorporate variables with more dimensions, include broader samples, conduct cross-cultural comparisons, and combine experimental designs or stratified analyses to further verify the mechanism and long-term effects of PE on alleviating SA through BI.

## 5 Conclusion

Based on a longitudinal cross-lagged model, this study explores the dynamic relationships among PE, BI, and SA, revealing both direct and indirect impacts of PE on mental health. The results show that PE can directly reduce SA and indirectly alleviate anxious emotions by improving BI. Adolescents who regularly engage in PE tend to have more positive BIs, and such positive perceptions enhance confidence in social situations, thereby reducing anxiety levels. The significant stability observed across the time dimension indicates that PE exerts a sustained effect on psychological adaptation and social function development.

## Data Availability

﻿The datasets presented in this study are included in the article and/or supplementary material. Further inquiries can be directed to the corresponding author.

## References

[ref1] Abdoli M. Scotto Rosato M. Desousa A. Cotrufo P. (2024). Cultural differences in body image: a systematic review. Soc. Sci. 13:305. doi: 10.3390/socsci13060305

[ref2] Alcaraz-Ibáñez M. Paterna A. Griffiths M. D. (2023). Social physical anxiety and eating disorders: a systematic review and meta-analysis. Body Image 45, 133–141﻿. doi: 10.1016/j.bodyim.2023.02.008 36871312

[ref3] Avramchuk O. (2018). Social anxiety disorder: relevance and perspectives. Psychosomatic Med. Gen. Pract. 3, e0303103–e0303103. doi: 10.26766/pmgp.v3i3.103

[ref4] Babic M. J. Morgan P. J. Plotnikoff R. C. Lonsdale C. White R. L. Lubans D. R. (2014). Physical activity and physical self-concept in youth: systematic review and Meta-analysis. Sports Med. 44, 1589–1601. doi: 10.1007/s40279-014-0229-z, 25053012

[ref5] Baribeau D. A. Vigod S. Brittain H. Vaillancourt T. Szatmari P. Pullenayegum E. (2022). Application of transactional (cross-lagged panel) models in mental health research: an introduction and review of methodological considerations. J. Can. Acad. Child Adolesc. Psychiatry 31, 124–134. 35919904 PMC9275371

[ref6] Beesdo K. Knappe S. Pine D. S. (2009). Anxiety and anxiety disorders in children and adolescents: developmental issues and implications for DSM-V. Psychiatr. Clin. North Am. 32, 483–524. doi: 10.1016/j.psc.2009.06.002, 19716988 PMC3018839

[ref7] Buchan M. C. Romano I. Butler A. Laxer R. E. Patte K. A. Leatherdale S. T. (2021). Bi-directional relationships between physical activity and mental health among a large sample of Canadian youth: a sex-stratified analysis of students in the COMPASS study. Int. J. Behav. Nutr. Phys. Act. 18:132. doi: 10.1186/s12966-021-01201-z, 34627283 PMC8501578

[ref8] Cannon C. J. Makol B. A. Keeley L. M. Qasmieh N. Okuno H. Racz S. J. . (2020). A paradigm for understanding adolescent social anxiety with unfamiliar peers: conceptual foundations and directions for future research. Clin. Child. Fam. Psychol. Rev. 23, 338–364. doi: 10.1007/s10567-020-00314-4, 32140896

[ref9] Cash T. F. Fleming E. C. (2002). The impact of body image experiences: development of the body image quality of life inventory. Int. J. Eat. Disord. 31, 455–460. doi: 10.1002/eat.10033, 11948650

[ref10] Chen S. Jing L. Li C. Wang H. (2024). Exploring the nexus between moderate-to-vigorous physical activity, self-disclosure, social anxiety, and adolescent social avoidance: insights from a cross-sectional study in Central China. Children 11:56. doi: 10.3390/children11010056 PMC10814873 38255369

[ref11] Chiu K. Clark D. M. Leigh E. (2021). Prospective associations between peer functioning and social anxiety in adolescents: a systematic review and meta-analysis. J. Affect. Disord. 279, 650–661. doi: 10.1016/j.jad.2020.10.055 33190116 PMC7758784

[ref12] Choukas-Bradley S. Roberts S. R. Maheux A. J. Nesi J. (2022). The perfect storm: a developmental–sociocultural framework for the role of social media in adolescent girls’ body image concerns and mental health. Clin. Child. Fam. Psychol. Rev. 25, 681–701. doi: 10.1007/s10567-022-00404-5, 35841501 PMC9287711

[ref13] Cohen S. (2014). Social supports and physical health: symptoms, health behaviors, and infectious disease[M]//life-span developmental psychology. Psychol. Press, 213–234.

[ref14] Deng Y. Wang X. (2024). The impact of physical activity on social anxiety among college students: the chain mediating effect of social support and psychological capital. Front. Psychol. 15:1406452. doi: 10.3389/fpsyg.2024.1406452 38957885 PMC11217649

[ref15] Dorčić T. M. Smojver-Ažić S. Božić I. Martinac Dorčić T. Malkoč I. (2023). Effects of social media social comparisons and identity processes on body image satisfaction in late adolescence. Eur. J. Psychol. 19:220. doi: 10.5964/ejop.9885, 37731891 PMC10508212

[ref16] Doré I. O’Loughlin J. L. Beauchamp G. Martineau M. Fournier L. (2016). Volume and social context of physical activity in association with mental health, anxiety and depression among youth. Prev. Med. 91, 344–350. doi: 10.1016/j.ypmed.2016.09.006 27609745

[ref17] Eime R. M. Young J. A. Harvey J. T. Charity M. J. Payne W. R. (2013). A systematic review of the psychological and social benefits of participation in sport for children and adolescents: informing development of a conceptual model of health through sport. Int. J. Behav. Nutr. Phys. Act. 10:98﻿. doi: 10.1186/1479-5868-10-98 23945179 PMC3751802

[ref18] Estrada-Tenorio S. Julián J. A. Aibar A. Martín-Albo J. Zaragoza J. (2020). Academic achievement and physical activity: the ideal relationship to promote a healthier lifestyle in adolescents. J. Phys. Act. Health 17, 525–532. doi: 10.1123/jpah.2019-0320, 32221041

[ref19] Ewuzie Z. Ezeano C. Aderinto N. (2024). A review of exercise interventions for reducing anxiety symptoms: insights and implications. Medicine 103:e40084. doi: 10.1097/MD.0000000000040084, 39465822 PMC11479437

[ref20] Feng W. Zhao L. Ge Z. Zhao X. Li T. Zhu Q. (2024). Association between physical activity and adolescent mental health in the post COVID-19: the chain mediating effect of self-esteem and social anxiety. PLoS One 19:e0301617. doi: 10.1371/journal.pone.0301617, 38758776 PMC11101116

[ref21] Field T. Diego M. Sanders C. E. (2001). EXERCISE is positively related to adolescents’ relationships and academics. Adolescence 36, 105–105. 11407627

[ref22] Frederick D. A. Reynolds T. A. (2022). The value of integrating evolutionary and sociocultural perspectives on body image. Arch. Sex. Behav. 51, 57–66. doi: 10.1007/s10508-021-01947-4, 33751287

[ref23] Gan L. Jiang Y. (2024). How is physical activity associated with social anxiety among college students? The mediating role of body image and the moderating role of self-esteem. Curr. Psychol. 43, 34679–34687. doi: 10.1007/s12144-024-06920-7

[ref24] Gunnell K. E. Flament M. F. Buchholz A. Henderson K. A. Obeid N. Schubert N. . (2016). Examining the bidirectional relationship between physical activity, screen time, and symptoms of anxiety and depression over time during adolescence. Prev. Med. 88, 147–152﻿. doi: 10.1016/j.ypmed.2016.04.002 27090920

[ref25] Hagströmer M. Oja P. Sjöström M. (2006). The international physical activity questionnaire (IPAQ): a study of concurrent and construct validity. Public Health Nutr. 9, 755–762. doi: 10.1079/PHN2005898, 16925881

[ref26] Hale G. E. Colquhoun L. Lancastle D. Lewis N. Tyson P. J. (2021). Physical activity interventions for the mental health and well-being of adolescents–a systematic review. Child Adolesc. Ment. Health 26, 357–368. doi: 10.1111/camh.12485, 34105239

[ref27] Hamaker E. L. Kuiper R. M. Grasman R. P. P. P. (2015). A critique of the cross-lagged panel model. Psychol. Methods 20, 102–116. doi: 10.1037/a0038889, 25822208

[ref28] Hamed M. Abbas H. S. (2019). Efficacy of psychodrama on social anxiety, self-esteem and psychological well-being of university students that met diagnosis of social anxiety disorder﻿. Knowledge & Research in Applied Psychology, 20, 22–30.

[ref29] Herring M. P. O’Connor P. J. Dishman R. K. (2010). The effect of exercise training on anxiety symptoms among patients: a systematic review. Arch. Intern. Med. 170, 321–331. doi: 10.1001/archinternmed.2009.530, 20177034

[ref30] Hoare E. Milton K. Foster C. Allender S. (2016). The associations between sedentary behaviour and mental health among adolescents: a systematic review. Int. J. Behav. Nutr. Phys. Act. 13:108. doi: 10.1186/s12966-016-0432-4, 27717387 PMC5055671

[ref31] Hollon S. D. Sexton T. L. (2012). Determining what works in depression treatment: translating research to relational practice using treatment guidelines. Couple Fam. Psychol. 1, 199–212. doi: 10.1037/a0029901

[ref32] Jazaieri H. Goldin P. R. Werner K. Ziv M. Gross J. J. (2012). A randomized trial of MBSR versus aerobic exercise for social anxiety disorder. J. Clin. Psychol. 68, 715–731. doi: 10.1002/jclp.21863, 22623316 PMC4136448

[ref33] Jia Z. (2025). An experimental study on the impact of social media images on users’ online social anxiety in China. Discov. Psychol. 5:14. doi: 10.1007/s44202-025-00337-4

[ref34] Jiang Y. Zhang B. Zhao H. (2025). Analysing the effect of physical exercise on social anxiety in college students using a chained mediation model. Sci. Rep. 15:2475. doi: 10.1038/s41598-025-87140-2, 39833375 PMC11756402

[ref35] Kajastus K. Kiviruusu O. Marttunen M. Ranta K. (2024). Associations of generalized anxiety and social anxiety symptoms with sleep duration, amount of intense exercise, and excessive internet use among adolescents. BMC Psychiatry 24:791. doi: 10.1186/s12888-024-06231-y, 39533195 PMC11559102

[ref36] King J. E. Jebeile H. Garnett S. P. Baur L. A. Paxton S. J. Gow M. L. (2020). Physical activity based pediatric obesity treatment, depression, self-esteem and body image: a systematic review with meta-analysis. Ment. Health Phys. Act. 19:100342. doi: 10.1016/j.mhpa.2020.100342

[ref37] Kline R. B. (2023). Principles and Practice of Structural Equation Modeling﻿. (5th ed.). New York, NY: Guilford publications.

[ref38] Kostanski M. Fisher A. Gullone E. (2004). Current conceptualisation of body image dissatisfaction: have we got it wrong? J. Child Psychol. Psychiatry 45, 1317–1325. doi: 10.1111/j.1469-7610.2004.00315.x, 15335351

[ref39] La Greca A. M. Lopez N. (1998). Social anxiety among adolescents: linkages with peer relations and friendships. J. Abnorm. Child Psychol. 26, 83–94. doi: 10.1023/a:1022684520514, 9634131

[ref40] Lin C. Y. Hsu S. H. J. Lee H. L. Wang C. Sung F. C. Su T. C. (2024). Examining a decade-long trend in exposure to per-and polyfluoroalkyl substances and their correlation with lipid profiles: insights from a prospective cohort study on the young Taiwanese population. Chemosphere 364:143072. doi: 10.1016/j.chemosphere.2024.143072 39128777

[ref41] Littleton H. (2008). ﻿Body image dissatisfaction: Normative discontent? Sex Roles, 59, 292–293. doi: 10.1007/s11199-008-9399-1﻿﻿﻿

[ref42] Liu F. Chen F. Li G. Li X. (2025). Factors influencing body image among Chinese secondary school students: a mixed methods approach. Psychol. Schs. 62, 1146–1157. doi: 10.1002/pits.23383

[ref43] Liu R. Hu H. Cao C. Han Y. Bai Y. Feng W. (2025). Sex differences in the relationship between body mass index in Chinese adolescents and future risk of hypertension: a decade-long cohort study. BMC Pediatr. 25:187. doi: 10.1186/s12887-025-05555-2, 40069609 PMC11900636

[ref44] Locke E. A. (1997). Self-efficacy: the exercise of control. Pers. Psychol. 50:801.

[ref45] Lubans D. Richards J. Hillman C. Faulkner G. Beauchamp M. Nilsson M. . (2016). Physical activity for cognitive and mental health in youth: a systematic review of mechanisms. Pediatrics 138:e20161642. doi: 10.1542/peds.2016-1642, 27542849

[ref46] Maes M. Nelemans S. A. Danneel S. Fernández-Castilla B. den Van Noortgate W. Goossens L. . (2019). Loneliness and social anxiety across childhood and adolescence: multilevel meta-analyses of cross-sectional and longitudinal associations. Dev. Psychol. 55, 1548–1565. doi: 10.1037/dev0000719, 30896228

[ref47] Marengo D. Longobardi C. Fabris M. A. Settanni M. (2018). Highly-visual social media and internalizing symptoms in adolescence: the mediating role of body image concerns. Comput. Hum. Behav. 82, 63–69. doi: 10.1016/j.chb.2018.01.003

[ref48] Miers A. C. Weeda W. D. Blöte A. W. Cramer A. O. J. Borsboom D. Westenberg P. M. (2020). A cross-sectional and longitudinal network analysis approach to understanding connections among social anxiety components in youth. J. Abnorm. Psychol. 129:82. doi: 10.1037/abn0000484, 31697140

[ref49] Moafi M. Bazzazian S. Amiri Majd M. (2019). The relationship between body image and social anxiety with positive and negative affect in the over-weight women. Rooyesh-e-Ravanshenasi J. 8, 123–132﻿. doi: 10.29252/rooyesh

[ref50] Neill R. D. Lloyd K. Best P. Tully M. A. (2020). The effects of interventions with physical activity components on adolescent mental health: systematic review and meta-analysis. Ment. Health Phys. Act. 19:100359. doi: 10.1016/j.mhpa.2020.100359

[ref51] Niven A. Fawkner S. Knowles A. M. Henretty J. Stephenson C. (2009). Social physique anxiety and physical activity in early adolescent girls: the influence of maturation and physical activity motives. J. Sports Sci. 27, 299–305. doi: 10.1080/02640410802578164, 19153865

[ref52] O’Dea J. A. (2004). Evidence for a self-esteem approach in the prevention of body image and eating problems among children and adolescents. Eat. Disord. 12, 225–239. doi: 10.1080/10640260490481438, 16864320

[ref53] O'gorman B. Sheffield J. Clarke R. Griffiths S. (2020). “Guys don't talk about their bodies”: a qualitative investigation of male body dissatisfaction and sociocultural influences in a sample of 40 Australian males. Clin. Psychol. 24, 123–132. doi: 10.1111/cp.12198

[ref54] Pilkionienė I. Širvinskienė G. Žemaitienė N. Jonynienė J. (2021). Social anxiety in 15–19 year adolescents in association with their subjective evaluation of mental and physical health. Children 8:737﻿. doi: 10.3390/children8090737 34572169 PMC8468452

[ref55] Plotnikoff R. C. Costigan S. A. Karunamuni N. Lubans D. R. (2013). Social cognitive theories used to explain physical activity behavior in adolescents: a systematic review and meta-analysis. Prev. Med. 56, 245–253. doi: 10.1016/j.ypmed.2013.01.013, 23370047

[ref56] Ren L. Xu Y. Guo X. Zhang J. Wang H. Lou X. . (2018). Body image as risk factor for emotional and behavioral problems among Chinese adolescents. BMC Public Health 18:1179. doi: 10.1186/s12889-018-6079-0 30326854 PMC6192148

[ref57] Revranche M. Biscond M. Husky M. M. (2022). Investigating the relationship between social media use and body image among adolescents: a systematic review. Encéphale 48, 206–218. doi: 10.1016/j.encep.2021.08.006 34801229

[ref58] Rodriguez-Ayllon M. Cadenas-Sánchez C. Estévez-López F. Muñoz N. E. Mora-Gonzalez J. Migueles J. H. . (2019). Role of physical activity and sedentary behavior in the mental health of preschoolers, children and adolescents: a systematic review and meta-analysis. Sports Med. 49, 1383–1410. doi: 10.1007/s40279-019-01099-5, 30993594

[ref59] Sabiston C. M. Pila E. Vani M. Thogersen-Ntoumani C. (2019). Body image, physical activity, and sport: a scoping review. Psychol. Sport Exerc. 42, 48–57﻿. doi: 10.1016/j.psychsport.2018.12.010

[ref60] Satorra A. Bentler P. M. (2001). A scaled difference chi-square test statistic for moment structure analysis. Psychometrika 66, 507–514. doi: 10.1007/bf02296192 PMC2905175 20640194

[ref61] Spence S. H. Rapee R. M. (2016). The etiology of social anxiety disorder: an evidence-based model. Behav. Res. Ther. 86, 50–67﻿. doi: 10.1016/j.brat.2016.06.007 27406470

[ref62] Stein M. B. Stein D. J. (2008). Social anxiety disorder. Lancet 371, 1115–1125. doi: 10.1016/S0140-6736(08)60488-2, 18374843

[ref63] Stojcic I. Dong X. Ren X. (2020). Body image and sociocultural predictors of body image dissatisfaction in Croatian and Chinese women. Front. Psychol. 11:731﻿. doi: 10.3389/fpsyg.2020.00731 32435214 PMC7218091

[ref64] Stonerock G. L. Hoffman B. M. Smith P. J. Blumenthal J. A. (2015). Exercise as treatment for anxiety: systematic review and analysis. Ann. Behav. Med. 49, 542–556. doi: 10.1007/s12160-014-9685-9, 25697132 PMC4498975

[ref65] Straatmann V. S. Almquist Y. B. Oliveira A. J. Veiga G. V. Rostila M. Lopes C. S. (2019). Stability and bidirectional relationship between physical activity and sedentary behaviours in Brazilian adolescents: longitudinal findings from a school cohort study. PLoS One 14:e0211470. doi: 10.1371/journal.pone.0211470, 30682158 PMC6347236

[ref66] Szuhany K. L. Sullivan A. J. Gills J. L. Kredlow M. A. (2025). The impact of exercise interventions on sleep in adult populations with depression, anxiety, or posttraumatic stress: review of the current evidence and future directions. J. Behav. Med. 48, 4–21. doi: 10.1007/s10865-024-00532-z, 39477903

[ref67] Teixeira D. S. Bastos V. Andrade A. J. Palmeira A. L. Ekkekakis P. (2024). Individualized pleasure-oriented exercise sessions, exercise frequency, and affective outcomes: a pragmatic randomized controlled trial. Int. J. Behav. Nutr. Phys. Act. 21:85. doi: 10.1186/s12966-024-01636-0, 39103923 PMC11299270

[ref68] Uchino B. N. Bowen K. Kent de Grey R. Mikel J. Fisher E. B. (2018). ﻿Social support and physical health: Models, mechanisms, and opportunities. In: Principles and Concepts of Behavioral Medicine. eds. Fisher E. B. Cameron L. D. Christensen A. J. Ehlert U. Guo Y. Oldenburg B. . New York, NY: Springer. 341–372. doi: 10.1007/978-0-387-93826-4_12﻿﻿

[ref69] Waaktaar T. Skaug E. Torgersen S. (2025). The etiological relationship between the general factors of psychopathology and personality; a longitudinal twin study from adolescence into young adulthood. Front. Psychol. 16:1564305. doi: 10.3389/fpsyg.2025.1564305 40697731 PMC12279783

[ref70] Walder N. Frey A. Berger T. Schmidt S. J. (2025). Digital mental health interventions for the prevention and treatment of social anxiety disorder in children, adolescents, and Young adults: systematic review and Meta-analysis of randomized controlled trials. J. Med. Internet Res. 27:e67067. doi: 10.2196/67067, 40504611 PMC12203032

[ref71] Wang L. Liu Y. (2022). “The effect of physical activity intervention on panic and anxiety symptoms in children, adolescents and early adulthoods: a Meta-analysis” in The Psychology of Panic﻿. ed. Motta R. W. (﻿London, UK: IntechOpen).﻿ doi: 10.5772/intechopen.106049

[ref72] Wang X. Lu C. Niu L. (2023). Body image construction and mental health levels among college students: a data survey of Chinese university students. Front. Public Health 11:1268775﻿. doi: 10.3389/fpubh.2023.1268775 37869184 PMC10585169

[ref73] Wang Q. Zainal Abidin N. E. Aman M. S. Wang N. Ma L. Liu P. (2024). Cultural moderation in sports impact: exploring sports-induced effects on educational progress, cognitive focus, and social development in Chinese higher education. BMC Psychol. 12:89. doi: 10.1186/s40359-024-01584-1, 38388547 PMC10885384

[ref74] Warburton D. E. R. Bredin S. S. D. (2017). Health benefits of physical activity: a systematic review of current systematic reviews. Curr. Opin. Cardiol. 32, 541–556. doi: 10.1097/HCO.0000000000000437, 28708630

[ref75] Wu J. Shao Y. Hu J. Zhao X. (2025). The impact of physical exercise on adolescent social anxiety: the serial mediating effects of sports self-efficacy and expressive suppression. BMC Sports Sci. Med. Rehabil. 17:57﻿. doi: 10.1186/s13102-025-01107-4 40121514 PMC11929206

[ref76] Wu J. Shao Y. Zang W. Hu J. (2025). Is physical exercise associated with reduced adolescent social anxiety mediated by psychological resilience?: evidence from a longitudinal multi-wave study in China. Child Adolesc. Psychiatry Ment. Health 19:17. doi: 10.1186/s13034-025-00867-8 40045423 PMC11884043

[ref77] Xian J. Zhang Y. Jiang B. (2024). Psychological interventions for social anxiety disorder in children and adolescents: a systematic review and network meta-analysis. J. Affect. Disord. 365, 614–627. doi: 10.1016/j.jad.2024.08.097 39173929

[ref78] Xiang G. Teng Z. Yang L. He Y. (2024). Longitudinal relationships among sociocultural pressure for body image, self-concept clarity, and emotional well-being in adolescents. J. Adolesc. 96, 98–111. doi: 10.1002/jad.12257, 37787102

[ref79] Yixin Z. Bin Z. Sicheng X. Zhuan L. Anqi Z. Chengwei Z. . (2024). The influence of mobile phone dependence on the development of social anxiety in junior high school students: longitudinal mediating effect of body shame. J. Psychol. Sci. 47:316. doi: 10.16719/j.cnki.1671-6981.20240208

[ref80] Zhang R. Liu F. Wang X. Wang S. (2024). Towards active health: a study on the relationship between physical activity and body image among college students. Heliyon 10:e38465. doi: 10.1016/j.heliyon.2024.e38465, 39391503 PMC11466608

[ref81] Zhao H. Chi Y. Shi L. (2025). Analyzing the effect of physical exercise on social anxiety in college students using the chain mediation model. Sci. Rep. 15:17751. doi: 10.1038/s41598-025-02445-6, 40404703 PMC12098904

[ref82] Zhao R. Kong X. Li M. Zhu X. Wang J. Ding W. . (2024). Shyness, sport engagement, and internalizing problems in Chinese children: the moderating role of class sport participation in a multi-level model. Behav. Sci. 14:661. doi: 10.3390/bs14080661 39199057 PMC11351716

[ref83] Zika M. A. Becker L. (2021). Physical activity as a treatment for social anxiety in clinical and non-clinical populations: a systematic review and three meta-analyses for different study designs. Front. Hum. Neurosci. 15:653108. doi: 10.3389/fnhum.2021.653108 34177489 PMC8230570

[ref84] Zuckerman S. L. Tang A. R. Richard K. E. Grisham C. J. Kuhn A. W. Bonfield C. M. . (2021). The behavioral, psychological, and social impacts of team sports: a systematic review and meta-analysis. Phys. Sportsmed. 49, 246–261. doi: 10.1080/00913847.2020.1850152, 33196337

